# Tobacco Transcription Factor *NtbHLH123* Confers Tolerance to Cold Stress by Regulating the *NtCBF* Pathway and Reactive Oxygen Species Homeostasis

**DOI:** 10.3389/fpls.2018.00381

**Published:** 2018-03-28

**Authors:** Qiang Zhao, Xiaohua Xiang, Dan Liu, Aiguo Yang, Yuanying Wang

**Affiliations:** ^1^Tobacco Research Institute, Chinese Academy of Agricultural Sciences, Qingdao, China; ^2^Hainan Cigar Institution, Haikou, China

**Keywords:** *NtbHLH123*, *NtCBF* pathway, reactive oxygen species (ROS), transcriptional regulation, cold stress, *Nicotiana tabacum*

## Abstract

Cold stress is a major environmental factor that impairs plant growth and development, geographic distribution, and crop productivity. The C-repeat binding factor (CBF) regulatory pathway has an essential role in response to cold stress. Here, we characterized a bHLH transcription factor from *Nicotiana tabacum*, *NtbHLH123*, in response to cold stress (4°C). Overexpression of *NtbHLH123* enhanced cold tolerance in transgenic tobacco plants. Based on yeast one-hybrid, chromatin immunoprecipitation PCR, and transient expression analysis assays, *NtbHLH123* binds directly to the G-box/E-box motifs in the promoter of the *NtCBF* genes and positively regulates their expression. Furthermore, *NtbHLH123*-overexpressing plants showed lower electrolyte leakage, reduced malondialdehyde contents, H_2_O_2_ and reactive oxygen species (ROS) accumulation under cold stress, which contributed to alleviating oxidative damage to the cell membrane after cold stress treatment. And *NtbHLH123* increased stress tolerance by improving the expression of a number of abiotic stress-responsive genes to mediate the ROS scavenging ability and other stress tolerance pathways. Taken together, we present a model suggesting that *NtbHLH123* is a transcriptional activator that functions as a positive regulator of cold tolerance by activating *NtCBF*, ROS scavenging-related, and stress-responsive genes.

## Introduction

Many environmental factors (e.g., high or low temperature, salt and drought) limit the growth and development, geographic distribution, yield, and quality of crop plants (Xiong et al., 2002). Low temperature (cold stress) is a key environmental stress (Chinnusamy et al., 2007). Cold stress affects physiological metabolic reactions, oxidative damage, poor germination, accelerated senescence, membrane damage, and tissue breakdown among others (Chinnusamy et al., 2007; Sanghera et al., 2011). Plants have developed sophisticated mechanisms to adapt to and tolerate cold stress by altering biochemical and physiological processes (Thomashow, 1999; Chinnusamy et al., 2007; Zhu, 2016). Many cold-responsive genes have been identified that are involved in the cold tolerance of plants, including phosphatases, protein kinases, transcription factors (TFs), and so on (Pearce, 1999; Shinozaki and Yamaguchi-Shinozaki, 2000). Among them, cold stress can rapidly induce the expression of many TFs, such as C-repeat-binding factor/dehydration-responsive binding protein (CBF/DREB), ABA-responsive element-binding protein/ABA-binding factor, APETALA2 (AP2)/ethylene responsive factor, basic region/leucine zipper motif (bZIP), MYB, basic helix-loop-helix (bHLH), and NAM, ATAF1, 2, and CUC2 (NAC) families, which in turn bind the promoter of numerous stress-responsive genes and regulate their expression (Chinnusamy et al., 2003; Vogel et al., 2005; Agarwal et al., 2006; Yamaguchi-Shinozaki and Shinozaki, 2006; Puranik et al., 2012; Shi et al., 2012).

The bHLH TF superfamily is a large group of functionally diverse proteins found in plants and animals (Garrell and Campuzano, 1991; Ledent and Vervoort, 2001). These proteins have a 60-amino-acid conserved domain, which contains two functionally distinct regions; a basic region containing 13–17 amino acids at the N-terminus that functions as a DNA-binding domain, and the HLH region at the C-terminus, which contributes to the formation of homodimers or heterodimers (Heim et al., 2003; Toledo-Ortiz et al., 2003). Research has demonstrated that plant bHLHs have important roles in regulating gene transcriptional networks involving light signaling (Pedmale et al., 2016), hormone signaling (Zhang et al., 2009), flavonoid biosynthesis (Xu et al., 2015), flowering time (Song et al., 2015), stomata, trichome and root hair formation (Feller et al., 2011), and abiotic stress responses including cold, heat (Chinnusamy et al., 2007; Shi et al., 2015; Zhu, 2016).

To date, 167 and 177 bHLH genes have been identified in *Arabidopsis* and rice, respectively (Mao et al., 2017). However, characterization of the functions of most bHLHs is relatively limited. Only 64 of 167 and 19 of 177 bHLHs have been characterized or partially characterized functionally in *Arabidopsis* and rice (Ji et al., 2016). For instance, *AtbHLH104*, *CmbHLH1*, and *MdbHLH104* enhance iron deficiency tolerance in *Arabidopsis*, chrysanthemum, and apple (Zhao et al., 2014, 2016b; Zhang et al., 2015). *MUTE*, *FAMA*, *SPEECHLESS*, *OsMUTE*, *OsFAMA*, *OsSPCH2*, and *ZmMUTE* regulate stomata differentiation in *Arabidopsis*, rice, and maize (Torii, 2015). *PIF1/PIL5*, *PIF3*, *PIF4*, *PIF5/PIL6*, and *OsbHLH102* are involved in light and gibberellin signaling in *Arabidopsis* and rice (Leivar and Monte, 2014). Recent studies have indicated that a number of bHLH genes are involved in abiotic stress responses. For example, *MYC2* functions as a TF in regulating wound, oxidative, drought, and salt stress responses (Fujita et al., 2006). *OsbHLH1* is involved in the cold signal transduction pathway (Wang et al., 2003). *PtrbHLH* has been shown to confer cold tolerance in *Poncirus trifoliata* (Huang et al., 2013). *OrbHLH2* overexpression improved tolerance to salt and osmotic stress in rice (Zhou et al., 2009). Overexpression of *OrbHLH001* confers freezing and salt tolerance (Li et al., 2010). *OsbHLH148* overexpression in rice increases drought tolerance (Seo et al., 2011). *AtbHLH112* is involved in abiotic stress tolerance (Liu et al., 2015). However, the functional roles of bHLH genes in response to cold stress have not been demonstrated in tobacco (*Nicotiana tabacum*).

Although several cold-related plant bHLH TFs have been characterized in *Arabidopsis*, rice, maize, *Pyrus ussuriensis*, *Populus suaveolens*, *Tamarix hispida*, and apple (Chinnusamy et al., 2007; Feng et al., 2012; Ji et al., 2016; Jin et al., 2016; Zhu, 2016). However, the functions of bHLH genes in cold tolerance remain poorly characterized in tobacco. As a model plant, tobacco has a key role in plant molecular research on cold tolerance and is sensitive to cold stress (Jin et al., 2017). In this study, we report the molecular cloning and functional characterization of *NtbHLH123* isolated from tobacco to identify and characterize the molecular mechanism of the tobacco response to cold stress. *NtbHLH123* was induced by cold stress (4°C), and overexpression in tobacco plants under the control of the cauliflower mosaic virus (CaMV) 35S promoter enhanced cold resistance. These data suggest that *NtbHLH123* has a positive role in cold resistance in tobacco, and may be an important candidate gene for the molecular breeding of cold-tolerant plants.

## Materials and Methods

### Plant Materials

*Nicotiana tabacum* L. ‘NC89’ was used as the wild type (WT). The seeds of the WT and transgenic lines were sterilized with 3.0% NaClO, and then germinated on plates containing Murashige and Skoog (MS) medium containing 3% sucrose and 0.6% agar at 25°C (Murashige and Skoog, 1962). The seedlings were transferred to soil and cultured in greenhouse (16-h light/8-h dark cycle at 25°C) and used for gene cloning, expressional analysis.

### Vector Construction and Genetic Transformation in Tobacco

To construct a reporter vector to analyze the expression of *NtbHLH123* in response to cold treatment, the full length of the promoter was amplified by PCR and cloned into pCXGUS-P to drive expression of the β-glucuronidase (GUS) reporter gene. For overexpression of *NtbHLH123* in tobacco, the *NtbHLH123* open reading frame (ORF) was cloned into the pRI 101-GFP vector containing a CaMV 35S promoter. These constructs were transferred into *Agrobacterium* strain LBA4404.

For tobacco transformation, the leaves of young seedlings from shoots grown *in vitro* were excised and cut into small strips. The leaf strips were immersed into *Agrobacterium* suspension culture for 15 min, and then dried with sterile filter paper. Then, the leaf strips were transferred onto MS medium with 0.1 mg L^-1^ 1-naphthaleneacetic acid (NAA) + 1.0 mg L^-1^ 6-benzylaminopurine (6-BA) for co-cultivation at 25 ± 1°C in the dark. A total of 3 days later, the leaf strips were subsequently transferred to selection medium containing 0.1 mg L^-1^ NAA + 1.0 mg L^-1^ 6-BA + 100 mg L^-1^ kanamycin + 250 mg L^-1^ carbenicillin. After 3 weeks, adventitious shoots were regenerated and transferred to rooting medium (1/2 MS + 150 mg L^-1^ kanamycin + 250 mg L^-1^ carbenicillin). Rooted plants were transplanted into soil. Homozygous seedlings were used for further investigation.

### Expression Analysis

For expression analysis, the uniformly sized seedlings were treated with control (25°C) or cold stress (4°C). Then the samples were collected from the various treated plants at specific time points and were immediately frozen in liquid nitrogen and stored at -80°C. Total RNA from the samples was extracted using TRIzol Reagent (Invitrogen, Carlsbad, CA, United States) following the manufacturer’s instructions. First-strand cDNA was synthesized using the PrimeScript First Strand cDNA Synthesis Kit transcriptase (TaKaRa, Dalian, China) according to manufacturer’s instructions.

Quantitative reverse transcription (qRT)-PCR was performed to determine transcript levels of *NtbHLH123* and stress-related genes in the transgenic and control plants. Reactions were carried out in a total volume of 20 μL containing 10 μL of SYBR Premix Ex Taq II, 0.8 μL of both forward and reverse primers, 0.4 μL of ROX Reference Dye II, and 100 ng of cDNA template. qRT-PCR reactions (95°C, 30 s; 95°C, 5 s; 60°C, 34 s; 40 cycles) were performed using the SYBR Green method on an Applied Biosystems 7500 Real-Time PCR System (Applied Biosystems, Foster City, CA, United States). Gene expression levels were calculated based on the full-quantification method, and *NtACTIN* (GenBank accession number: U60495) was used as the internal control for normalizing gene expression. All of the primers used are shown in Supplementary Table S1.

### Protein Extraction and Western Blot

Total proteins of WT and three transgenic plants were extracted in 1 × SDS buffer. For Western blot, the proteins were separated with SDS–PAGE gel and transferred onto PVDF membrane (Roche, United States). The gel blot was probed with anti-GFP (Beyotime, China) and the signals were visualized by chemiluminescence using the ECL plus kit (Millipore, Bedford, MA, United States) according to the manufacturer’s instructions. Anti-Actin served as a loading control.

### Cold Tolerance Analysis

For germination rate analysis, the aseptic seeds of control and three transgenic lines were planted on MS medium, and were incubated for 8 days under 25 ± 1 or 4 ± 1°C. Changes of germination (testa rupture) were measured.

For the root length measurements, 10-days-old seedlings were maintained at 25 ± 1 or 4 ± 1°C. The treatment plates were placed vertically. The relative root length was calculated as follows: elongation of root length/original of root length.

For adult plant analysis, the seedlings were first transplanted into plastic containers filled with a mixture of soil and sand (1:1) and grown under normal conditions. The 40-days-old tobacco plants of WT and transgenic plants were directly exposed to cold stress (4°C) for 2 days without cold acclimation, and then moved to recover under normal conditions for 14 days. All the results are based on the average of three independent biological replicates.

### Yeast One-Hybrid Assay

The yeast one-hybrid (Y1H) assay was performed using the Matchmaker One-Hybrid Library and Screening Kit (Clontech, Mountain View, CA, United States). The full length of the *NtbHLH123* gene was amplified from tobacco cDNA using PCR and recombined into the pGADT7 vector (Clontech, Mountain View, CA, United States). *NtCBF* promoter fragments and mutated fragments were amplified from tobacco genomic DNA and cloned into the pAbAi vector (Clontech, Mountain View, CA, United States). Both the pAbAi bait vector and the pGADT7 prey vector were introduced into Y1H Gold Yeast (Clontech, Mountain View, CA, United States) and cultured on SD/–Leu medium with or without 150 ng mL^-1^ aureobasidin A (AbA) for 3–5 days at 30°C. The primer sequences used are listed in Supplementary Table S2.

### Chromatin Immunoprecipitation (ChIP)-PCR Analysis

ChIP analysis was carried out using the Chromatin Immunoprecipitation Assay Kit (Millipore, MA, United States) following the manufacturer’s instructions. Protein-DNA complexes were cross-linked and incubated with GFP antibody (Beyotime, China). IP protein-DNA complexes were precipitated with Protein A-Sepharose beads overnight at 4°C. The DNA fragments in the IP complex were purified as described by Zhao et al. (2016a). DNA fragment enrichment was analyzed using qRT-PCR. The primers used are listed in Supplementary Table S2.

### Transient Expression Assay in Tobacco Leaves

The *NtbHLH123* coding region was inserted into pGreenII 62-SK to generate effector plasmids. The promoter fragments and mutant promoter fragments were cloned into pGreenII 0800-LUC to create reporter plasmids. The vectors of the effector and reporter were separately transformed into *Agrobacterium tumefaciens* LBA4404 cells, and the transformed LBA4404 cells were used to infiltrate the leaves of *Nicotiana benthamiana* for transient expression, which was sampled after 2 days. Firefly (LUC) and *Renilla* (REN) luciferase were detected with dual luciferase assay reagents (Promega, Madison, WI, United States) using an Infinite M200 plate reader (Tecan, Männedorf, Switzerland). The Promoter activities were calculated as the ratio of *LUC*/*REN*.

### GUS Staining and Activity Analysis

The GUS staining was carried out according to the method of Zhao et al. (2016b). Transgenic tobacco plants were immersed into GUS staining buffer at 37°C for 4 h in the dark. After staining, the transgenic tobacco plants were de-stained and photographed.

For quantitative analysis of GUS activity, fluorescence was measured using a VersaFluor spectrofluorometer (excitation wavelength of 365 nm and emission wavelength of 450 nm).

### Histochemical Staining Analysis

The H_2_O_2_ accumulation in leaves was stained by 3,3′-diaminobenzidine (DAB). Details of assay have been described by Zhao et al. (2016a).

The reactive oxygen species (ROS) accumulation was detected using the fluorescent probe 2′,7′-dichlorodihydrofluorescein diacetate (DCFH2-DA, Sigma-Aldrich, United States) according to Zhao et al. (2016a). For DCFH2-DA staining, leaves were treated with 10 μM DCFH2-DA for 20 min and then washed with distilled H_2_O. The images were obtained using the confocal microscope (E_x_, 488 nm; E_m_, 522 nm) (Leica Microsystems, Wetzlar, Germany).

### Physiological Measurements

The leaves, which were exposed to control or cold stress conditions, were sampled from the WT and three transgenic plants (L1, L2, and L3). For electrolyte leakage (EL) measurement, the leaves were placed in 30 ml distilled water and shaken at room temperature for 3 h, the initial conductivity (E1) was measured with electric conductivity meter. Then the leaves were boiled for 20 min and were cooled to room temperature, the electrolyte conductivity (E2) was measured, and the electrolyte leakage (%) was calculated as follows: Electrolyte leakage (%) = 100 × E1/E2. The malondialdehyde (MDA) accumulation was measured using the thiobarbituric acid–based method with an MDA assay kit (A003-3, Jiancheng, Nanjing, China). The Chlorophyll content was measured as described by Feng et al. (2012).

For the enzyme assays, the SOD activities were determined by measuring the inhibiting rate of the enzyme to O_2_^-⋅^ produced by the xanthine morpholine with xanthine oxidase using the SOD Detection Kit (A001, Jiancheng, Nanjing, China). The CAT activity was measured as the degradation of H_2_O_2_ at 405 nm according to the CAT Detection Kit (A007, Jiancheng, Nanjing, China). The reaction mixture, which contained 0.1 mM H_2_O_2_ and 60 mM potassium phosphate buffer (pH 7.0), was incubated at 37°C for 1 min. Then, the reaction was stopped by adding 32.5 mM ammonium molybdate, and the yellow complex of H_2_O_2_ and ammonium molybdate was detected at 405 nm. The POD activity was measured based on the change of absorbance at 420 nm by catalyzing H_2_O_2_ according to POD Detection Kit for plant (A084-3, Jiancheng, Nanjing, China).

### Statistical Analysis

Cold stress treatment of the WT and transgenic lines was repeated at least three independent biological replicates with consistent results. All experimental data are averages of at least three independent biological replicates. The data were analyzed by Duncan’s multiple range tests in the ANOVA program of SPSS (IBM SPSS 22), taking ^∗^*P* < 0.01, ^∗∗^*P* < 0.001 as significantly different.

## Results

### *NtbHLH123* Transcription Is Induced by Cold Stress

Transcriptome analysis, which was conducted using cDNA samples extracted from tobacco seedlings treated with or without cold stress, showed that *NtbHLH123* was upregulated by cold treatment (Jin et al., 2017), suggesting that *NtbHLH123* might be a cold-responsive bHLH TF gene. Bioinformatics analysis showed that *NtbHLH123*, which has seven exons and six introns and is localized on the chromosome 1 of the tobacco genome, contains a 1389-bp ORF and encodes 463 amino acids with a predicted molecular weight of 50.72 kDa and a pI of 6.40 (Supplementary Figure S1A). Motif analysis showed that *NtbHLH123* has a highly conserved bHLH domain containing 61 amino acids in its C-terminal regions (Supplementary Figure S1B).

**FIGURE 1:**
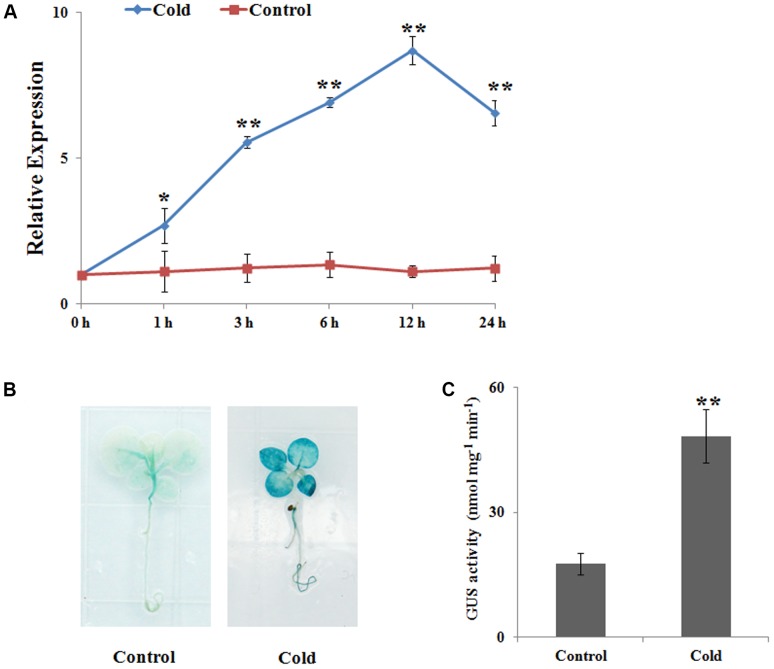
*NtbHLH123* expression profiles in response to cold stress. **(A)** Analysis of *NtbHLH123* expression using real-time PCR. Tobacco plants were subjected to 4°C for the indicated times. The relative expression levels were normalized to the expression of the *NtACTIN* gene. Data are the means ± SD of three independent biological replicates. **(B,C)** Analysis of *NtbHLH123* expression in response to cold treatment using **(B)** β-glucuronidase (GUS) histochemical staining and **(C)** GUS activity measurement. The *Pro_NtbHLH123_::GUS* transgenic seedlings were grown in Murashige and Skoog medium with or without treatment at 4°C for 4 h, and GUS histochemical staining and GUS activity measurement were performed. Data are expressed as the mean ± SD as determined from three independent biological replicates. Asterisks indicate that the value is significantly different from that of the control at the same time point (^∗∗^*P* < 0.001).

To examine *NtbHLH123* transcript levels in response to cold stress, we performed qRT-PCR analysis. *NtbHLH123* expression changed slightly during the studied period under normal condition; however, under cold stress (4°C), *NtbHLH123* expression was highly induced and reached a peak (eightfold) at 12 h, and decreased at 24 h (**Figure 1A**). In addition, GUS staining and GUS activity showed that *NtbHLH123* expression increased in both the leaves and roots of plants expressing Pro*_NtbHLH123_*::GUS in response to cold stress (**Figures 1B,C**). These results suggest that cold stress activates *NtbHLH123* transcription in tobacco.

### *NtbHLH123* Overexpression Increases Cold Tolerance

**FIGURE 2:**
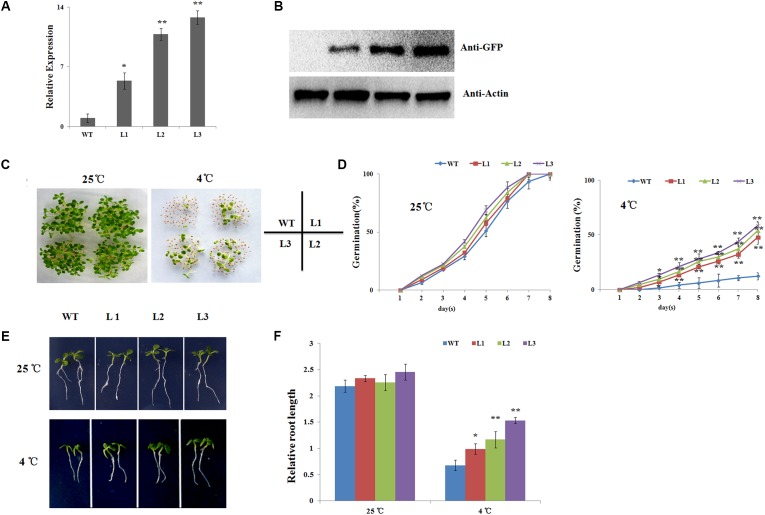
Seed germination and seedling root lengths of *NtbHLH123* transgenic tobacco were insensitive to cold stress. **(A)** *NtbHLH123* transcript levels in transgenic tobacco. WT, wild-type; L1, L2, and L3, transgenic tobacco lines. **(B)** NtbHLH123-GFP fusion protein levels in *35S::NtbHLH123-GFP* transgenic plants as determined by immunoblot analysis using an anti-GFP antibody. Anti-actin antibody was used as a loading control. **(C,D)** Seed germination rates were determined in the overexpression lines and WT. All tests were repeated at least three independent biological replicates, and approximately 50 seeds were counted for each experiment. Data are expressed as the means ± SD. **(E,F)** Comparison of transgenic and WT plant growth on plates. Tobacco seedlings were grown vertically for 8 days and the root lengths were measured under normal or cold-stress conditions. Data are expressed as the mean ± SD as determined from three independent biological replicates of 30 seedlings. Asterisks indicate that the value is significantly different from that of the WT at the same time point (^∗^*P* < 0.01; ^∗∗^*P* < 0.001).

To investigate the function of *NtbHLH123*, *35S::NtbHLH123* transgenic tobacco plants were obtained via *Agrobacterium*-mediated transformation. In total, eight T0 independent transgenic lines were confirmed by PCR with CaMV 35S and *NPTII* primers (Supplementary Figure S2). Among them, three independent lines (L1, L2, and L3) were used for functional characterization, while the WT was used as a control. qRT-PCR and Western blot assays indicated that all three transgenic tobacco lines had increased transcript levels and produced more NtbHLH123-GFP fusion protein (**Figures 2A,B**), indicating that *NtbHLH123* was overexpressed in these transgenic tobacco lines.

To examine whether *NtbHLH123* has a role in the response to cold stress, the seeds of transgenic tobacco plants and the WT control were sown on MS medium and exposed to 25 or 4°C, and the germination rates were examined. At 25°C condition, the transgenic and WT seeds have the same germination rates and all seeds germinated at 8 days. At 4°C condition, seed germination of both the WT and the transgenic lines was inhibited. However, 47.7–58.5% of the three transgenic lines seeds germinated, while only 12.3% of the WT seeds germinated, indicating that overexpression of *NtbHLH123* in tobacco leads to reduced sensitivity of seed germination to cold stress (**Figures 2C,D**). Moreover, we measured the relative root lengths after cold treatment. At 25°C condition, there is not much difference in the relative root lengths of three transgenic seedlings (2.21, 2.32, and 2.36) and WT control (2.18) (**Figures 2E,F**). In contrast, the relative root lengths of three transgenic seedlings were 1.01, 1.16, and 1.53, respectively, while that of WT control was 0.58 at 4°C condition (**Figures 2E,F**). Therefore, *NtbHLH123* overexpression enhanced cold tolerance in transgenic tobacco seedlings.

**FIGURE 3:**
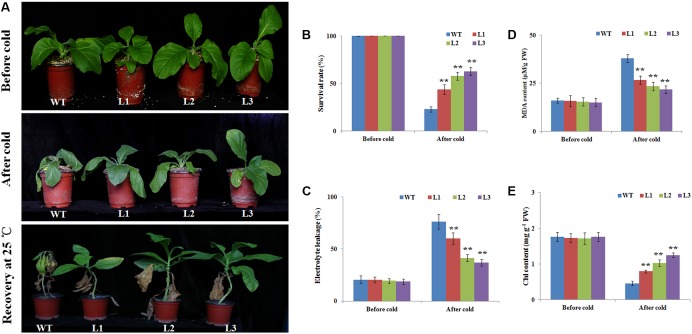
Overexpression of *NtbHLH123* enhances cold tolerance in transgenic tobacco. **(A)** Phenotypes of 40-days-old seedlings of wild type (WT) and transgenic plants subjected to cold treatment (4°C) 2 days followed by recovery at 25°C for 14 days. At least three independent biological replicates were performed for each individual experiment. **(B)** Survival ratios of the WT and transgenic plants after recovery from cold stress treatment. Data represent the means ± SD of at least three independent biological replicates. Asterisks indicate that the value is significantly different from that of the WT at the same time point (^∗^*P* < 0.01; ^∗∗^*P* < 0.001). **(C,D)** Malondialdehyde (MDA) content and electrolyte leakage, respectively. FW, fresh weight. Data represent the means ± SD of at least three independent biological replicates. Asterisks indicate that the value is significantly different from that of the WT at the same time point (^∗^*P* < 0.01; ^∗∗^*P* < 0.001). **(E)** Effects of cold stress on the chlorophyll (chl) content in WT and transgenic tobacco leaves. Data are the means ± SD of three independent biological replicates. Asterisks indicate that the value is significantly different from that of the WT at the same time point (^∗∗^*P* < 0.001).

Adult plants (40-days-old seedlings) were also used to assess cold stress tolerance. Before cold treatment, there were no morphological differences between the transgenic and WT plants (**Figure 3A**). When seedlings were exposed to cold stress (4°C) for 2 days, cold injuries were observed in the WT plants more serious than the transgenic plants. After recovery for 14 days under 25°C, the survival rates of the WT plants was 10%, while the transgenic plants were 39.3, 50.1, and 59.6%, respectively (**Figures 3A,B**). EL and MDA levels, important indicators of cell damage, can reflect the extent of membrane injure (Huang et al., 2013). The EL in the WT plants (63.1%) was significantly higher than in three transgenic plants (49.9, 38.8, and 33.4%) (**Figure 3C**). While the MDA content in the WT were 1.47–1.81 times higher than in the transgenic plants (**Figure 3D**), suggesting that membrane damage was alleviated in the transgenic plants under cold stress condition. We also quantitatively measured the chlorophyll levels. After the cold treatment, the chlorophyll contents of L1 (0.79 mg g^-1^ FW), L2 (1.02 mg g^-1^ FW), and L3 (1.24 mg g^-1^ FW) transgenic plants were higher than the WT plants (0.46 mg g^-1^ FW) (**Figure 3E**). Overall, the results indicated that *NtbHLH123* overexpression in tobacco resulted in increased protection of membrane integrity and enhanced cold tolerance compared with the WT control.

Next, the transcript levels in both transgenic plants and WT were analyzed under control and cold stress (Supplementary Figure S3). The relative mRNA levels of *NtbHLH123* in the transgenic lines were more than twofold, significantly higher than that of WT under cold stress condition. These results indicate that the transgenic plants improve the cold stress tolerant, mainly through the overexpression of *NtbHLH123* in tobacco.

### *NtbHLH123* Directly Regulates *NtCBF* Gene Expression

**FIGURE 4:**
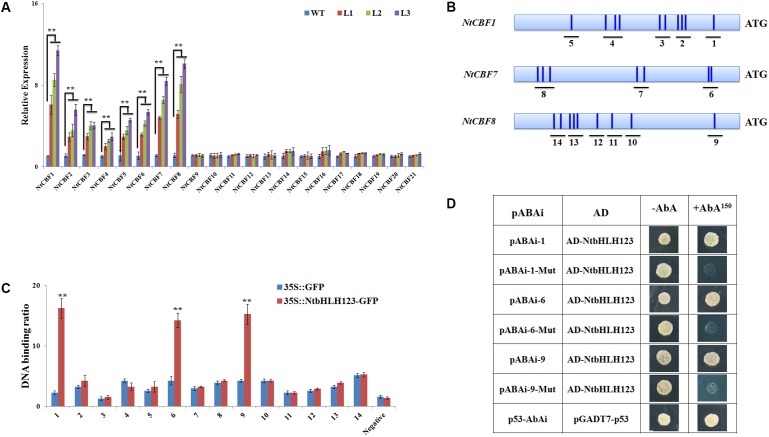
*NtbHLH123* binds to the promoters of *NtCBF* genes. **(A)** Expression analysis of *NtCBF* genes in the wild type (WT) and three transgenic tobacco lines after cold treatment. Data are the means ± SD of three independent biological replicates. The value for the WT control was set to 1. Asterisks indicate that the value is significantly different from that of the WT at the same time point (^∗∗^*P* < 0.001). **(B)** Schematic diagrams of the *NtCBF1*, *NtCBF7*, and *NtCBF8* promoter regions showing the presence of E-box DNA motifs. Transverse lines show the positions of primers used in the chromatin immunoprecipitation (ChIP)-PCR experiment. **(C)** ChIP-qPCR analyses of the DNA binding ratio of *NtbHLH123* to the promoters of *NtCBF1*, *NtCBF7*, and *NtCBF8*. Data represent the means ± SD of three independent biological replicates. Asterisks indicate that the value is significantly different from that of the *35S::GFP* at the same time point (^∗∗^*P* < 0.001). **(D)** Analysis of the binding of *NtbHLH123* to the promoters of *NtCBF1*, *NtCBF7*, and *NtCBF8* based on a yeast one-hybrid study. The normal or mutant promoters co-transformed with AD-NtbHLH123 and the positive controls were grown on selection medium with or without 150 ng mL^-1^ aureobasidin A (AbA).

Using the Tobacco Genomic Database^1^, we identified 21 *NtCBF* genes (Supplementary Figure S4A). A phylogenetic tree indicated that the 21 *NtCBF* genes were closely related to four *Arabidopsis AtCBF* genes (AP2 family) (Supplementary Figure S4B). To determine whether the *NtbHLH123*-regulated plant response to cold stress is dependent on the CBF pathway, we examined the expression levels of *NtCBF* genes in *NtbHLH123* transgenic tobacco plants after cold treatment using qRT-PCR. *NtbHLH123* overexpression significantly induced the expression of *NtCBF1–NtCBF8* genes (**Figure 4A**). These results demonstrated that *NtbHLH123* positively regulates plant cold tolerance by upregulating *NtCBF* expression.

In our previous works, *NtbHLH123* proteins specifically bind to the E-box and G-box sequence ‘CANNTG’ (unpublished data). Using the plantCARE and PLACE programs, we found many G-box or E-box motifs in the promoters of the *NtCBF1–NtCBF8* genes (**Figure 4B**). To detect the binding ability of *NtbHLH123* to the *NtCBF* promoters, we performed ChIP-qPCR using *35S::GFP* and *35S::NtbHLH123-GFP* transgenic plants. After immunoprecipitation with anti-GFP, the DNA fragments containing G-box or E-box motifs were amplified with qRT-PCR. A clear band was detected around 1 (*NtCBF1*), 6 (*NtCBF7*), and 9 (*NtCBF8*) when NtbHLH123-GFP was precipitated (**Figure 4C**), demonstrating that *NtCBF1*, *NtCBF7*, and *NtCBF8* are target genes of *NtbHLH123*. In addition, E-box elements were present in other *NtCBF* genes (*NtCBF2–NtCBF6*). However, the ChIP-PCR results showed that none of these recruited MdbHLH104-GFP proteins (Supplementary Figure S5). The Y1H assay was used to confirm the results of the ChIP-qPCR assay. The *NtbHLH123* ORF and promoter fragments containing the E-box motifs were cloned into plasmids pGADT7 and pAbAi, respectively. pGADT7-*NtbHLH123* and pAbAi-1/6/9 were co-transformed into Y1H Gold, which were selected on SD/–Leu medium with or without 150 ng mL^-1^ AbA. When 150 ng mL^-1^ AbA was added, only the cells co-transformed with pGADT7-*NtbHLH123* and pAbAi-1/6/9 grew (**Figure 4D**). These results indicate that the *NtbHLH123* protein can bind to the E-box elements in the *NtCBF* promoters.

**FIGURE 5:**
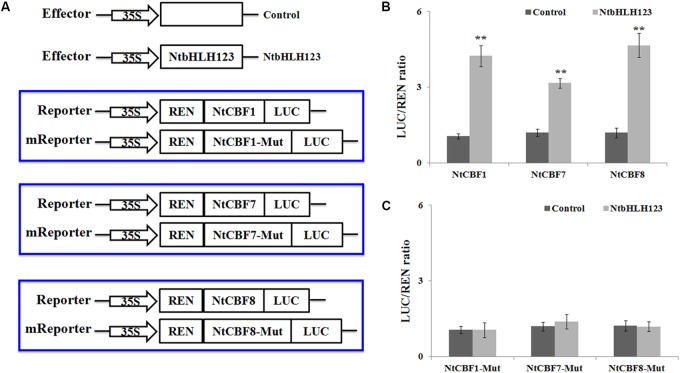
*NtbHLH123* directly activates the expression of *NtCBF* genes. **(A)** Illustration of the effector and reporter constructs used for transient expression assay. The mReporter vector was constructed using the mutated E-box motifs. **(B,C)** Transient expression assay of the promoter activity in tobacco leaves co-transformed with the effector and reporter constructed using the normal or mutated E-box motif. Data represent the means ± SD of three independent biological replicates. Asterisks indicate that the value is significantly different from that of the control at the same time point (^∗∗^*P* < 0.001).

We performed transient expression assays to examine how *NtbHLH123* regulates the expression of *NtCBFs* (*NtCBF1*, *NtCBF7*, and *NtCBF8*). The *NtbHLH123* ORF was cloned into pGreenII 62-SK, and the of the *NtCBF1*, *NtCBF7*, and *NtCBF8* promoter fragments were cloned into pGreenII 0800-LUC (**Figure 5A**). A significant increase in luminescence intensity was observed when *NtbHLH123* was co-expressed with the *NtCBF1*, *NtCBF7*, or *NtCBF8* promoters, whereas 35Spro:*NtbHLH123* failed to activate *NtCBF1*, *NtCBF7*, or *NtCBF8* expression when the E-box motifs were mutated (**Figure 5B**). These findings reveal that *NtbHLH123* can activate *NtCBF1*, *NtCBF7*, and *NtCBF8* expression.

### Analysis of ROS Levels, and Antioxidant Enzyme Activities

**FIGURE 6:**
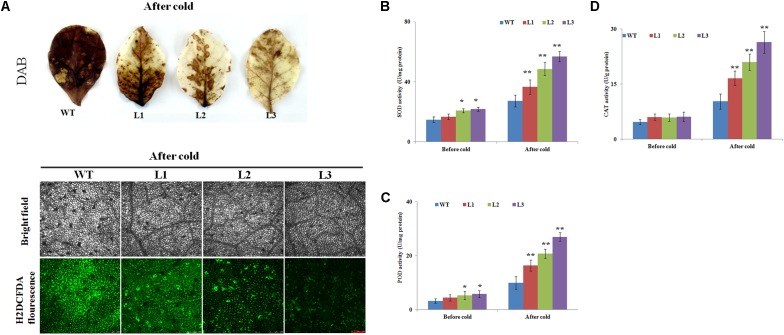
Histochemical staining analysis and activities of antioxidant enzymes in wild type (WT) and transgenic plants before and after cold treatment. **(A)** H_2_O_2_ and ROS accumulation in transgenic and WT plants based on histochemical staining before and after cold treatment. **(B–D)** Activities of **(B)** superoxide dismutase (SOD), **(C)** peroxidase (POD), and **(D)** catalase (CAT) in the WT and transgenic plants under normal or cold stress conditions. Data represent the means ± SD of at least three independent biological replicates. Asterisks indicate that the value is significantly different from that of the WT at the same time point (^∗^*P* < 0.01; ^∗∗^*P* < 0.001).

It is well documented that cold stresses usually results in the excessive accumulation of ROS (e.g., O_2_^-^ or H_2_O_2_), which can be used as indicators of cell damage (Mittler, 2002). To test whether overexpression of *NtbHLH123* genes also affects ROS levels, leaves from WT plants and transgenic plants were stained with DAB or H2DCFDA after treatment with cold stress. Histochemical staining indicated that WT plants showed higher level of ROS than transgenic plants, after the plants were subjected to cold stress (**Figure 6A**).

The above results showed that the WT plants had higher ROS levels than the transgenic plants; therefore, three key antioxidant enzymes (e.g., CAT, POD, and SOD), which play critical roles in ROS scavenging and affect cellular ROS levels (Mittler, 2002), were measured before and after cold stress treatment. The transgenic plants showed slightly higher activities of these enzymes than the WT plants before cold treatment (**Figures 6B–D**). After cold treatment, SOD activities were markedly enhanced in the transgenic lines, whereas the WT plants showed only a slight increase (**Figure 6B**). Exposure to cold condition resulted in notably rise of POD activity in transgenic lines, which was 1.65-, 2.09-, and 3.11-fold of that in WT, respectively (**Figure 6C**). The CAT activities of the transgenic plants (between 16.5 U g^-1^ protein and 26.3 U g^-1^ protein) were significantly higher than WT (10.2 U g^-1^ protein) (**Figure 6D**).

### Expression Analysis of ROS Scavenging-Related and Stress-Responsive Genes in Transgenic and WT Plants Before and After Cold Treatment

**FIGURE 7:**
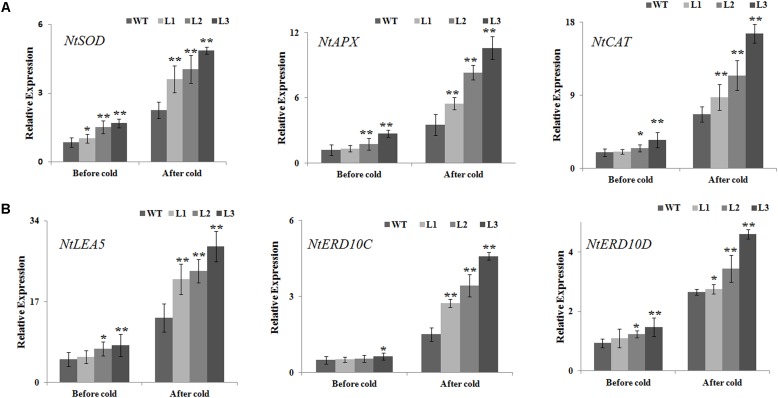
Analysis of relative gene expression of reactive oxidation species scavenging-related **(A)** and stress-responsive genes **(B)** based on quantitative reverse transcription PCR in WT and transgenic plants before and after cold treatment. Data represent the means ± SD of at least three independent biological replicates. Asterisks indicate that the value is significantly different from that of the WT at the same time point (^∗^*P* < 0.01; ^∗∗^*P* < 0.001).

The positive effect of *NtbHLH123* overexpression on antioxidant enzyme activities suggested that *NtbHLH123* might be involved in the regulation of ROS homeostasis under cold stress treatment. Therefore, the expression levels of ROS-related genes encoding enzymes for direct ROS detoxification were detected in the WT and transgenic plants before and after cold treatment. The transcript levels of *NtAPX*, *NtSOD*, and *NtCAT* were slightly enhanced in the transgenic lines compared with those of WT in the absence of cold stress. However, all three genes were dramatically upregulated by 1.5- to 5-fold in the *NtbHLH123*-overexpressing plants after cold treatment (**Figure 7A**), suggesting that *NtbHLH123* may be a key regulator upstream of some ROS-related genes, and overexpression of *NtbHLH123* could trigger the induction of a series of ROS-related genes to cope positively with adverse environmental conditions. In addition, we analyzed the transcript levels of several cold stress defensive proteins (*NtLEA5*, *NtERD10C*, and *NtERD10D*). Under normal conditions, the mRNA levels of all three genes were slightly higher in the transgenic plants than in the WT plants. After the cold treatment, the expression levels of three genes increased rapidly in the WT and transgenic plants, but the *NtLEA5* and *NtERD10C* caused a 2- to 5-fold increase in transgenic plants and *NtERD10D* resulted only in a 0.8- to 1.7-fold upregulation transgenic plants compared with the WT (**Figure 7B**). These results suggest that *NtbHLH123* overexpression enhances the mRNA levels of ROS scavenging-related and stress-responsive genes with cold stress treatment.

## Discussion

Cold stress is one of the major environmental factors which restricts the growth, development, productivity, and distribution of crops worldwide. Cold tolerance is the major mechanism by which plants adapt to stress through the regulation of a wide range of cold-responsive genes (Thomashow, 1999). Numerous studies have indicated that TFs have significant roles in regulating various biological processes to protect plant cells against cold-induced damage (Yamaguchi-Shinozaki and Shinozaki, 2006; Chinnusamy et al., 2007; Shi et al., 2015; Zhu, 2016). Among them, bHLH TFs contain a conserved bHLH domain, which involved in DNA binding through sequence-specific interactions in the promoter regions of target genes (Carretero-Paulet et al., 2010). However, only a few bHLH proteins have been characterized involving in abiotic stress in plants, especially in tobacco. Here, *NtbHLH123* was separated from tobacco based on its differential expression in response to stresses treatment (unpublished data). *NtbHLH123* was found to have a highly conserved bHLH domain of bHLH proteins, suggesting that it is a putative tobacco bHLH protein (Supplementary Figure S1). In addition, *NtbHLH123* expression was induced by cold stress (**Figure 1**), suggesting that *NtbHLH123* may have a regulatory role during cold stress.

Studies have shown that the overexpression of ICE1, which results in insensitivity to chilling and freezing stresses, positively regulates *CBF* gene expression under cold stress (Chinnusamy et al., 2003; Budhagatapalli et al., 2016). In our work, the stress cold tolerance assay showed that the transgenic plants exhibited enhanced tolerance to cold stress compared with the WT, by measuring survival rate, EL (%), MDA, and chlorophyll contents, in accordance with phenotypic observation, suggesting that overexpression of *NtbHLH123* conferred cold stress. However, it is worth mentioning that the transcript level of *NtbHLH123* is induced by cold stress in both WT and transgenic plants. The possible explanation for this is that the *NtbHLH123* transgenic lines exhibited high levels than in the WT under normal condition, so the overexpression plants could ensure a rapid response by plants to resist the cold stress under non-cold-acclimated conditions. While the resistance of WT is gradually enhanced, it was cold damage in the early stages of responses to cold stress. In addition, the transcript levels of *NtbHLH123* were induced in WT, while was lower than the transgenic plants upon exposure to cold stress (Supplementary Figure S3), it may be because of CaMV 35S promoter can be induced by some stresses (Czechowski et al., 2005; Wang et al., 2010; Jin et al., 2016). In addition, *CBF* genes are induced by cold, and by overexpression enhance cold tolerance in transgenic *Arabidopsis*, rice, tobacco, rapeseed, tomato, and apple (Gilmour et al., 2000; Hsieh et al., 2002; Ito et al., 2006; Yang et al., 2011). The ICE1-CBF-COR transcriptional cascade is the best-understood cold acclimation signaling pathway (Chinnusamy et al., 2007). In this pathway, CBFs/DREBs are activated by cold and directly regulate the expression of cold-responsive genes by binding to their promoter regions (Thomashow, 1999, 2010; Chinnusamy et al., 2007). Recent evidence has indicated that CBF-dependent pathways are mediated by many protein kinases or TFs at transcriptional, posttranslational, and posttranscriptional levels (Shi et al., 2015). In *Arabidopsis*, AtICE1 and AtICE2, respectively, bind the E-box (or G-box) motifs in the *AtCBF3* and *AtCBF1* promoters to trigger expression (Chinnusamy et al., 2003; Lee et al., 2005; Fursova et al., 2009). Here, *NtbHLH123* overexpression resulted in the elevated expression of *NtCBF* genes (**Figure 4A**), suggesting that *NtbHLH123* may act as a signal transduction component in a potential CBF-dependent pathway and is associated with cold tolerance in tobacco. ChIP-PCR and Y1H assays indicated that *NtbHLH123* protein could bind to the E-box motifs of *NtCBFs* promoters, further determining that it acts as transcriptional activator (**Figures 4**, **5**). However, in tobacco, *NtbHLH123* only bound to the promoters of the *NtCBF1*, *NtCBF7*, and *NtCBF8* genes (**Figure 4** and Supplementary Figure S5). In conformity to the specific binding, the increases in expression were observed for *NtCBF1*, *NtCBF7*, and *NtCBF8*, although *NtCBF2*, *NtCBF3*, *NtCBF4*, *NtCBF5*, and *NtCBF6* expression was also enhanced (**Figure 4A**). This may be explained either by indirect regulation of the expression of *NtCBF* genes by *NtbHLH123* or by regulation of the expression of other *NtCBF* genes by *NtCBF1*, *NtCBF7*, and *NtCBF8*, as is the case for *AtCBFs* in *Arabidopsis* (Novillo et al., 2004).

Several reports have shown that ROS (e.g., O_2_^-^ or H_2_O_2_) production is triggered following exposure to abiotic stresses (Suzuki and Mittler, 2006; Suzuki et al., 2012). When plants are exposed to cold stress, high ROS levels result in oxidative stress, leading to lipid peroxidation and membrane damage in plants (Mittler, 2002; Choudhury et al., 2017). Therefore, plants have evolved complex mechanisms to scavenge the overproduction of ROS and adapt to low-temperature-induced damage. MDA and EL are related to the membrane system (Huang et al., 2013). In this study, higher MDA and EL levels in the WT plants implied that they might have been subjected to more oxidative stress than the *35S::NtbHLH123* transgenic plants (**Figures 3C,D**). Furthermore, the WT plants exhibited more intense histochemical staining compared with the transgenic plants after cold stress (**Figure 6A**), suggestive of less ROS accumulation in the transgenic plants than the WT plants. However, ROS accumulation relies greatly on ROS-scavenging systems under different abiotic stress conditions (Mittler et al., 2004; Choudhury et al., 2017). To reduce the influence of oxidative stress, antioxidant enzymes (e.g., SOD, CAT, and POD) have essential roles in maintaining ROS homeostasis in plants (Mittler, 2002, 2017; Suzuki and Mittler, 2006). The activities of SOD, POD, and CAT in the transgenic lines were slightly higher in the transgenic plants than in the WT under normal conditions (**Figures 6B–D**), which this may be due to the fact that remaining a basal level of ROS in cells for life and oxidative stress was not serious in WT and transgenic plants (Mittler, 2017). However, SOD, CAT, and POD activities were significantly higher in the transgenic lines than in the WT after cold stress (**Figures 6B–D**). In line with the antioxidant enzyme activities, ROS-scavenging-related genes (e.g., *NtSOD*, *NtCAT*, and *NtAPX*) were expressed at higher levels in the *NtbHLH123*-overexpressing plants than in the WT plants before and after cold stress (**Figure 7A**). The expression levels of these genes were consistent with the higher activities of antioxidant enzymes described above. These results suggest that overexpressing *NtbHLH123* could increase cold tolerance, partially through a better ROS-scavenging system.

Furthermore, the mRNA levels of stress-responsive genes (i.e., *NtLEA5*, *NtERD10C*, and *NtERD10D*) were analyzed using qRT-PCR before and after cold treatment. In previous studies, most of these genes or their homologs have been shown to respond to abiotic stresses (Hundertmark and Hincha, 2008; Jin et al., 2016). Stronger induction of these genes was detected in the transgenic plants than in the WT plants, suggestive of less damage to transgenic plants, which was supported by the lower amount of membrane damage under cold stress (**Figure 7B**). In the future, additional research is needed to determine whether *NtbHLH123* directly regulates stress-responsive genes to enhance cold tolerance.

## Conclusion

**FIGURE 8:**
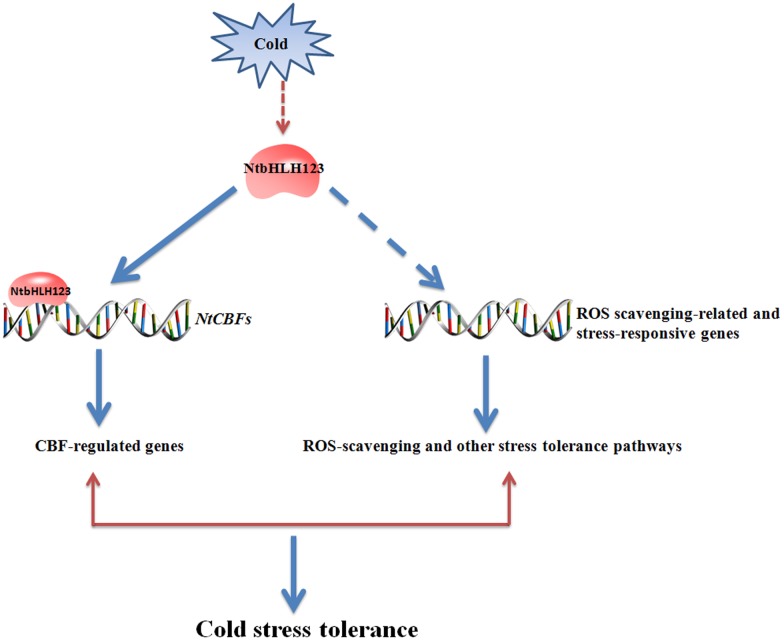
Model of the regulatory network of *NtbHLH123* involved in the cold stress response. Cold stress activates the expression of *NtbHLH123*. Cold-activated *NtbHLH123* subsequently binds to E-box (or G-box) motifs to regulate the expression of its target genes (e.g., *NtCBFs*) or regulates reactive oxidative species (ROS) scavenging-related and stress-responsive genes, leading to improved cold stress tolerance.

We characterized a TF, *NtbHLH123*, which functions as a positive regulator to activate stress-responsive gene expression, conferring cold tolerance in a CBF-dependent manner and resulting in increased cold tolerance in tobacco (**Figure 8**). These results provide new information that helps to clarify the complex CBF-dependent pathway of transcriptional control upon cold stress in plants. Moreover, *NtbHLH123* may be a valuable gene candidate for cold-tolerance trait improvement.

## Author Contributions

QZ and YW conceived and designed the research. QZ performed most of the experiments. QZ, XX, DL, AY, and YW performed the research. QZ and YW analyzed the data and wrote the paper.

## Conflict of Interest Statement

The authors declare that the research was conducted in the absence of any commercial or financial relationships that could be construed as a potential conflict of interest. The reviewer SS and handling Editor declared their shared affiliation.

## Footnotes

1 http://solgenomics.net/
