# Supplementary material for: Tobacco Transcription Factor NtbHLH123 Confers Tolerance to Cold Stress by Regulating the NtCBF Pathway and Reactive Oxygen Species Homeostasis
Source: Front Plant Sci. 2018 Mar 28;9:381. doi: 10.3389/fpls.2018.00381 (PMC5882786; doi:10.3389/fpls.2018.00381)
Supplement: Supplementary file 1 [file Data_Sheet_1.DOCX]

**The Title:**

Tobacco transcription factor *NtbHLH123* confers tolerance to cold stress by regulating the *NtCBF* pathway and reactive oxygen species homeostasis

**The full names of all the authors:**

Qiang Zhao^1^*, Xiaohua Xiang^2^, Dan Liu^1^, Aiguo Yang^1^, Yuanying Wang^1^*

**The names and address of the institution:**

^1^Tobacco Research Institute, Chinese Academy of Agricultural Sciences, Qingdao 266101, Shandong Province, China **;**

^2^Hainan Cigar Institution, Haikou, Hainan Province, China **;**


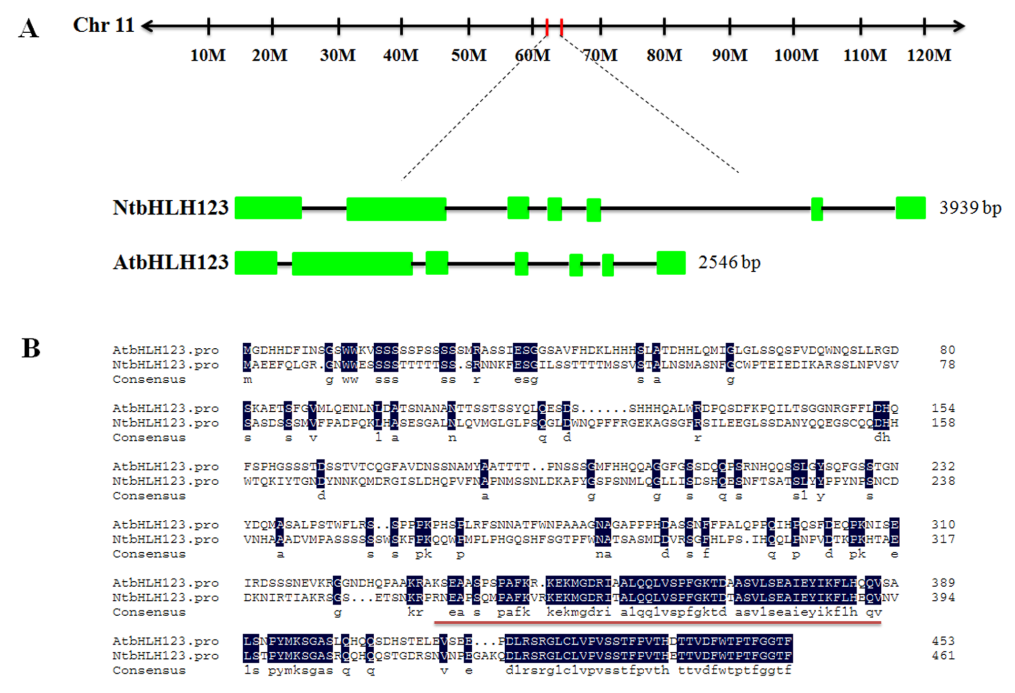


**Figure S1.** Genomic structure and sequence analysis of *NtbHLH123*.

(A) Genomic structure of *NtbHLH123* and *AtbHLH123*.

(B) Sequence alignment and overall domain structure of NtbHLH123 and AtbHLH123. A putative bHLH domain region is indicated. Identical residues are shown in blue.

**
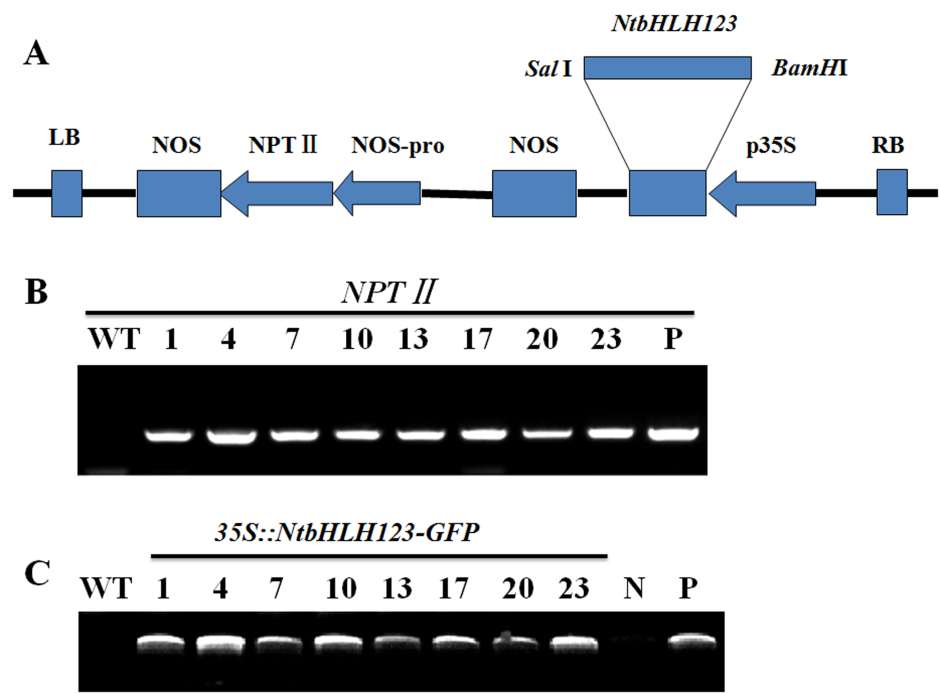
**

**Figure S2.** Overexpression construct and identification of *35::NtbHLH123* transgenic tobacco plants.

(A) Schematic diagram of the *NtbHLH123* overexpression construct used for the tobacco transformation. LB, left border; NOS, nopaline synthase terminator; *NPTII*, neomycin phosphotransferase II; 35S, cauliflower mosaic virus 35S promoter; RB, right border.

(B) PCR confirmation of kanamycin-resistant plants using *NPTII*-specific primers. P, plasmid DNA (positive control); WT, wild type. The numbers indicate different transgenic lines.

(C) PCR confirmation of kanamycin-resistant plants using CaMV35S-*NtbHLH123* primers. P, plasmid DNA (positive control); WT, wild type. The numbers indicate different transgenic lines.


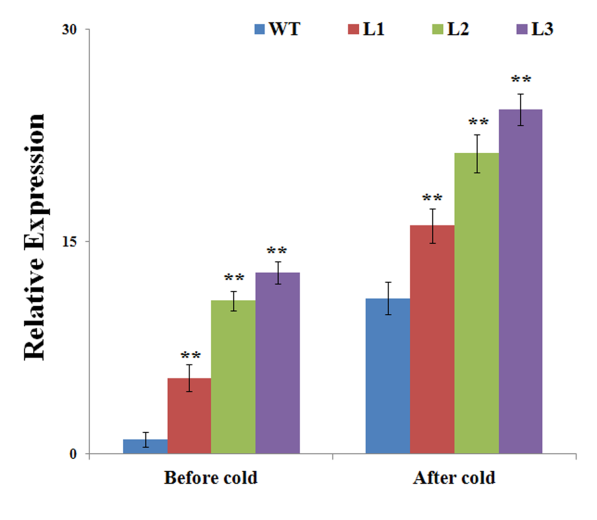


**Figure S**3. Expression levels of NtbHLH123 before and after cold stress using qRT-PCR. Asterisks indicate a significant difference between transcript levels of NtbHLH123 in transgenic lines at the same time point. (**P<0.001).

**
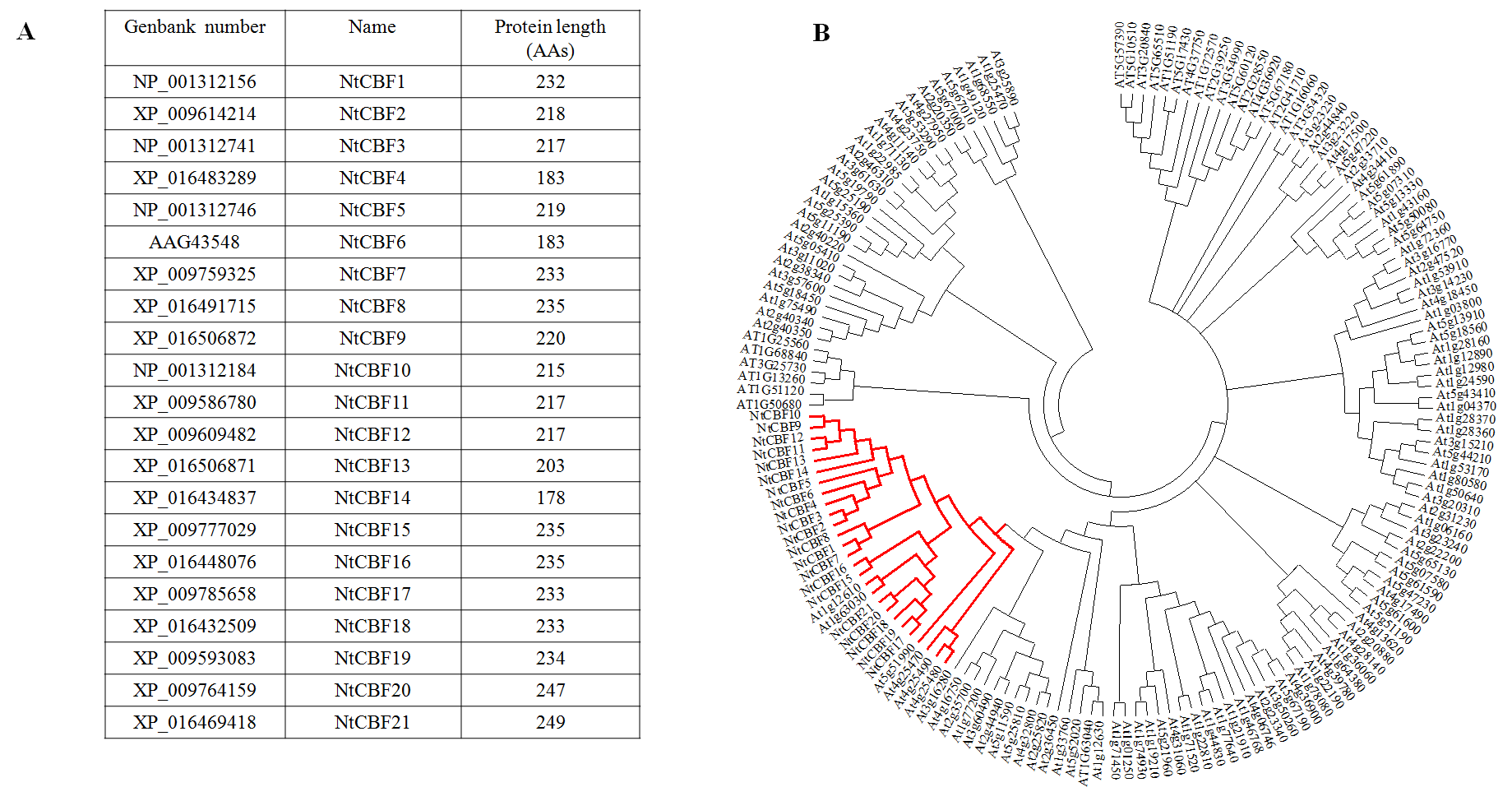
**

**Figure S4.** *NtCBF* genes identified in tobacco. NtCBFs and AtCBFs are shown in red.

(A) The predicted C-repeat binding factor proteins in tobacco were named NtCBF1–NtCBF21.

(B) Phylogenetic tree constructed using NtCBFs and *Arabidopsis* bHLHs. The predicted full-length amino acid sequences of 21 *NtCBF* and 146 *Arabidopsis* *AP2* genes were aligned using Clustal X ver. 1.83 software and the phylogenetic tree was constructed using MEGA 4.0 software using the neighbor-joining method with 1,000 bootstrap replicates.


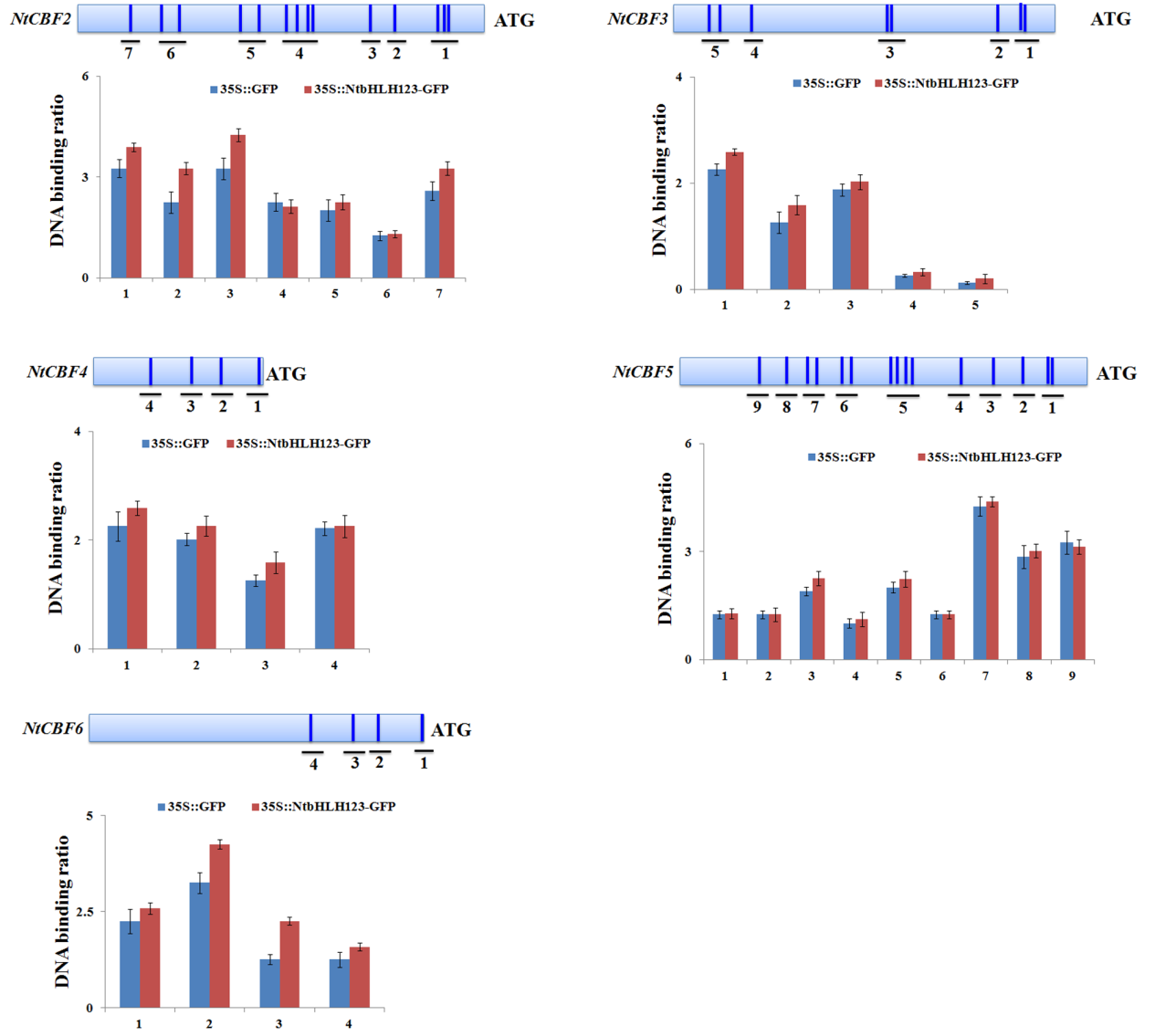


**Figure S**5. Chromatin immunoprecipitation (ChIP)-qPCR analysis of the DNA binding ratio of NtbHLH123 to the *NtCBF2*, *NtCBF3*, *NtCBF4*, *NtCBF5*, and *NtCBF6* promoters in transgenic tobacco plants containing either the empty vector (*35S::GFP*) or the NtbHLH123 gene (*35S::NtbHLH123-GFP*). Black lines represent the positions of primers used in the ChIP-PCR experiment. The data represent the means ± SD of three independent biological replicates.

**Table S1** Primers used in real time RT-PCR

| Gene |  | | Primers |
| --- | --- | --- | --- |
| *NtActin* |  | | 5’-CTATTCTCCGCTTTGGACTTGGCA-3’ |
|  |  |  | 5’-ACCTGCTGGAAGGTGCTGAGGGAA-3’ |
| *NtSOD* |  | | 5’-CTCCTACCGTCGCCAAAT-3’ |
|  |  |  | 5’-GCCCAACCAAGAGAACCC-3’ |
| *NtCAT* | | | 5’-AGGTACCGCTCATTCACACC-3’ |
|  |  |  | 5’-AAGCAAGCTTTTGACCCAGA-3’ |
| *NtAPX* | |  | 5’-CAAATGTAAGAGGAAACTCAGAGGA-3’ |
|  |  |  | 5’-CAGCCTTGAGCCTCATGGTACCG-3’ |
| *NtPOD* | | | 5’-GCTGTTCGACGAGTTGTTAACAG-3’ |
|  |  |  | 5’-CTCTGGCTGAGTTGTTGTTGG-3’ |
| *NtbHLH123* | | | 5’-GGTTGTTGGCCGACAGAAAT-3’ |
|  |  |  | 5’-TTTTCACCGCGGAAGAAAGG-3’ |
| *NtCBF1* | | | 5’-GGTTACCCATCCCGACTTCA-3’ |
|  |  |  | 5’-TCTCGGCATGCAGAAAAGTG-3’ |
| *NtCBF2* | | | 5’-TTTACTCGGACCCACTTGCT-3’ |
|  |  |  | 5’-TCAGAAACCCACTTGCCTGA-3’ |
| *NtCBF3* | | | 5’-TGCTTGGAAGTTGCCTGTTC-3’ |
|  |  |  | 5’-AGAATAGCGCCTCCTCATCC-3’ |
| *NtCBF4* | | | 5’-GGATATTCAGAAAGCGGCGG-3’ |
|  |  |  | 5’-TTCGCTAGTAATCCCGGCAT-3’ |
| *NtCBF5* | | | 5’-GCCTATTCCAGCTTCAACCG-3’ |
|  |  |  | 5’-AGAATAGCGCCTCCTCATCC-3’ |
| *NtCBF6* | | | 5’-GGATATTCAGAAAGCGGCGG-3’ |
|  |  |  | 5’-TTCGCTAGTAATCCCGGCAT-3’ |
| *NtCBF7* | | | 5’-CAATACCAGCTTCATCGGCC-3’ |
|  |  |  | 5’-GCAAGTAATCTCGGCATGCA-3’ |
| *NtCBF8* | | | 5’-GGTTACCCATCCCGACTTCA-3’ |
|  |  |  | 5’-TCTCGGCATGCAGAAAAGTG-3’ |
| *NtCBF9* | | | 5’-GGTCGTTCTGCTTGCTTGAA-3’ |
|  |  |  | 5’-CGCCATACTCGCTAGCAATC-3’ |
| *NtCBF10* | | | 5’-CGTCATGCAACATACAGGGG-3’ |
|  |  |  | 5’-TTCAAGCAAGCAGAACGACC-3’ |
| *NtCBF11* | | | 5’-ACAGCAGCTCCCCATGTAAT-3’ |
|  |  |  | 5’-TGGGGTAAGTTCCAAGCCAT-3’ |
| *NtCBF12* | | | 5’-ATGGCTTGGAACTTACCCCA-3’ |
|  |  |  | 5’-GCCAGCACATGATTCTCCTG-3’ |
| *NtCBF13* | | | 5’-GACGTGGCGACTATTGCATT-3’ |
|  |  |  | 5’-ACTGATCAGATGGGCGGAAA-3’ |
| *NtCBF14* | | | 5’-GGTCGTTCTGCTTGCTTGAA-3’ |
|  |  |  | 5’-AAGAGCGCTTCCTCATCCAT-3’ |
| *NtCBF15* | | | 5’-GCGGCTCTTCAAGTTTCCAA-3’ |
|  |  |  | 5’-GCCTCTTTTCATAGCGGGTG-3’ |
| *NtCBF16* | | | 5’-GACATTCAAATCGCGGCTCT-3’ |
|  |  |  | 5’-AGCATTCCTTCCGCCATACT-3’ |
| *NtCBF17* | | | 5’-TTCTGGCCAGGAGAGGAAAC-3’ |
|  |  |  | 5’-TTGTCTGCCATTCTTGCGTC-3’ |
| *NtCBF18* | | | 5’-GGGGAGTGAGAAGGAGGAAC-3’ |
|  |  |  | 5’-AAGCAGAGTCCGCGAAATTC-3’ |
| *NtCBF19* | | | 5’-ACTGGCAATGGTGATGAGGA-3’ |
|  |  |  | 5’-CATCCCAGCTGAAACAACCC-3’ |
| *NtCBF20* | | | 5’-AGCACCCGACGAAGGAATTA-3’ |
|  |  |  | 5’-CTGCTTACTAGGCTCGCGTA-3’ |
| *NtCBF21* | | | 5’-TTTCGTCCGGATCCTGAGTT-3’ |
|  |  |  | 5’-TTTCTTGCCACGATCCCTCT-3’ |
| *NtLEA5* | | | 5’-TTGAATCTGGGGTTTTGGTT-3’ |
|  |  |  | 5’-GGAAGCATTGACGAGCTAGG-3’ |
| *NtERD10C* | | | 5’-AACGTGGAGGCTACAGATCG-3’ |
|  |  |  | 5’-GTTCCTCTTGGGCATGAGTT-3’ |
| *NtERD10D* | | | 5’-GAGGACACGGCTGTACCAGT-3’ |
|  |  |  | 5’-GCGCCACTTCCTCTGTCTT-3’ |

**Table S2** Primers used for ChIP-PCR assays and Y1H constructs

| Gene |  | | Primers |
| --- | --- | --- | --- |
| NtCBF1-1 | | | CCCAGTGTCCAGTAAAACCC |
|  |  |  | AGTAATGGGGCCTAGCTACT |
| NtCBF1-2 | |  | CCAAACAACCCGCCTCTTTA |
|  |  |  | GCGAGATTTGCCCCAATTGA |
| NtCBF1-3 | | | TTGCTTATCCCATGAATCAAA |
|  |  |  | TACATCGGAGGCCTTTGAAC |
| NtCBF1-4 | | | CGTGCGTCTAATCACTTGTCA |
|  |  |  | CCACGTGTCACTGCCTTATG |
| NtCBF1-5 | | | GGCCGCACTTCTTCATATTC |
|  |  |  | AGAGATGGATTCTGGGGTCA |
| NtCBF7-6 | | | TCCGTGCCCAAATAGGTTTA |
|  |  |  | TGGGGCCTAGCTACTTTATGA |
| NtCBF7-7 | | | GCTGCTCTCTTTTCCTTTGC |
|  |  |  | GCACTCACCCACGATTTTCT |
| NtCBF7-8 | | | CTCATGCCACTAGTCCAGCA |
|  |  |  | CCCAAGTTCATCTGTTTTGGA |
| NtCBF8-9 | | | GGTACCCCCAGTGTCCAGTA |
|  |  |  | GAAAAAGTAATGGGGCCTAGC |
| NtCBF8-10 | | | GCCCTACTCGGTGTTCAAAA |
|  |  |  | TTACAAAGTCTGCCGAGAGGA |
| NtCBF8-11 | | | TCCTCCTCCATACCCTCAAA |
|  |  |  | CCATTGAGTGCACAATAGCA |
| NtCBF8-12 | | | TCCAAGCAATATGGTTCAAGC |
|  |  |  | TTTGAGGGTATGGAGGAGGA |
| NtCBF8-13 | | | ACCCGCCTCTTTATCCATCT |
|  |  |  | AGTGCCCCCTTAGGACAAGT |
| NtCBF8-14 | | | AACAATCAAGGCAACCAAGG |
|  |  |  | GGATAAAGAGGCGGGTTGTT |
| NtCBF2-1 | | | ACTAACAAGTTGCCGCATCC |
|  |  |  | ACACGGATTTGAAGGGTGAT |
| NtCBF2-2 | | | TGGTTCCCCAAAGATTCACT |
|  |  |  | TTGCTACCACCACTGACCAA |
| NtCBF2-3 | | | GCCTAATCAAAATGAATCACAGAA |
|  |  |  | CAAGTCAAAGATCAGCAAGCA |
| NtCBF2-4 | | | CCCTACGAATCCTTGGACCT |
|  |  |  | GACCGACTCCAAATGCAAAT |
| NtCBF2-5 | | | TGTTAATTAGCGGAGTTCAAAGG |
|  |  |  | TCCATGTGTAACATCTCGTCCT |
| NtCBF2-6 | | | AAATTTCAAAATAGGGGAAAAACA |
|  |  |  | TTTGAAAAACTCCAAAAAGCTG |
| NtCBF2-7 | | | TGCTAATCCAAGTCCAGTAGAAGA |
|  |  |  | GCAAATACGAGGTTGCCATT |
| NtCBF3-1 | | | CTAACAAGTTGCCGCATCAG |
|  |  |  | CGCGGAGTTAGAGGGTGATA |
| NtCBF3-2 | | | ACCATATATCATTTTCAAATGTCAAG |
|  |  |  | TCTTCCTTCCTTCCTTTTTCG |
| NtCBF3-3 | | | TCTGTGTTTATGCATTTATTTATGG |
|  |  |  | CCACAAACCCCATATCCATT |
| NtCBF3-4 | | | CGATTCAGCCAAAAATTGCT |
|  |  |  | ATGTTTTCTTTCCGCGTTTG |
| NtCBF3-5 | | | GATGAGTTGAAAAAGAGTTTTGGA |
|  |  |  | AAACATTGTGAGTATGAACC |
| NtCBF4-1 | | | GCCCACTTTTCACATTGACC |
|  |  |  | GAGATGAAGAATCAGCAAGTGG |
| NtCBF4-2 | | | ATGACAAGTTGCCGCATCAC |
|  |  |  | GCGGGGTGATAAGGAGACAG |
| NtCBF4-3 | | | ATTCATGCCGACCTTTGACT |
|  |  |  | CAACTTGTCATGGGCCTTTC |
| NtCBF4-4 | | | TGTTTCACTAATTGTCCTGCAA |
|  |  |  | TTTGTTATTGTCACTCCCCAAA |
| NtCBF5-1 | | | AGTAACAAGTTGCCGCATCC |
|  |  |  | ACACGGAGTTGAAGGGTGAT |
| NtCBF5-2 | | | TTTGACAGCTACCGGCAAAT |
|  |  |  | TGGGACTACACTTTGGCTACG |
| NtCBF5-3 | | | CCTCTAAAAGATCAAGTTGGCATAA |
|  |  |  | AAGCATTTCCGAAGGCTAAT |
| NtCBF5-4 | | | ACAACTTGTTTGACACTCCAAA |
|  |  |  | CTTTAAAGTTGTTTTTCCATTCCAA |
| NtCBF5-5 | | | ATCCTGGACCTGGAAATGTG |
|  |  |  | GCCTTTCGAGTTTCCTCTTG |
| NtCBF5-6 | | | GAGATTTGCTTCTTCAATTGTGTG |
|  |  |  | TGGTGGATGTCTACTCTTCTTCC |
| NtCBF5-7 | | | CCATGAGATGATATGTATGTCTGC |
|  |  |  | TTGCTTTTATTTCTTGTTCATGTTTT |
| NtCBF5-8 | | | ACCATGACATGCCTCATATCAAC |
|  |  |  | CGTATGCACACAACTTCATACCA |
| NtCBF5-9 | | | TTTTCTTCAAGATGATTTATGCTTC |
|  |  |  | TCTTAAATATGTCCTACCGCTTTT |
| NtCBF6-1 | | | TCTGAAGTGATCAAGAATAATGGA |
|  |  |  | CATCAGAAAGGTTAGCTCTGTCA |
| NtCBF6-2 | | | CATCACCAGAAACACACACG |
|  |  |  | TGAAACTAAAGAAGTTTGGAACG |
| NtCBF6-3 | | | ATTCATGCCGACCTTTGACT |
|  |  |  | TGGGCCTTTCTACTTTCTTCC |
| NtCBF6-4 | | | CTGCAATATGGCCCTACGAC |
|  |  |  | TGCGATGCATTTTTAATTGG |
